# Gypenoside induces apoptosis by inhibiting the PI3K/AKT/mTOR pathway and enhances T-cell antitumor immunity by inhibiting PD-L1 in gastric cancer

**DOI:** 10.3389/fphar.2024.1243353

**Published:** 2024-02-28

**Authors:** Hongliang Wu, Wenjing Lai, Qiaoling Wang, Qiang Zhou, Rong Zhang, Yu Zhao

**Affiliations:** ^1^ Department of Pharmacy, The University-Town Hospital of Chongqing Medical University, Chongqing, China; ^2^ Department of Pharmacy, The Second Affiliated Hospital of Army Medical University, Chongqing, China

**Keywords:** gypenoside, gastric cancer, PI3K/Akt/mTOR pathway, PD-L1, antitumor immunity

## Abstract

**Introduction:** Gypenoside is a natural extract of *Gynostemma pentaphyllum* (Thunb.) Makino, a plant in the Cucurbitaceae family. It has been reported to have antitumor effects on the proliferation, migration and apoptosis of various types of cancer cells. However, the use of gypenoside in the treatment of gastric cancer has not been studied. In the present study, we explored the therapeutic effect of gypenoside on gastric cancer and the potential molecular mechanism.

**Methods and Results:** Our results showed that gypenoside induced apoptosis in HGC-27 and SGC-7901 cells in a time-dependent and dose-dependent manner. Network pharmacology analyses predicted that gypenoside exerts its therapeutic effects through the PI3K/AKT/mTOR signaling pathway. Furthermore, molecular docking and western blot experiments confirmed that gypenoside induced the apoptosis of gastric cancer cells through the PI3K/AKT/mTOR signaling pathway. In addition, network pharmacological analysis revealed that the common targets of gypenoside in gastric cancer were enriched in the immune effector process, PD-L1 expression, the PD-1 checkpoint pathway, and the Jak-STAT signaling pathway. Furthermore, molecular docking and western blot assays demonstrated that gypenoside could bind to STAT3 and reduce its phosphorylation. Thus, the transcription of PD-L1 was inhibited in gastric cancer cells. Moreover, coculture experiments of gastric cancer cells with gypenoside and primary mouse CD8^+^ T cells showed that gastric cancer cells treated with gypenoside could enhance the antitumor ability of T cells. Animal experiments confirmed the antitumor effect of gypenoside, and the expression of PD-L1 was significantly downregulated in the gypenoside-treated group.

**Conclusion:** Gypenoside induced the apoptosis of gastric cancer cells by inhibiting the PI3K/AKT/mTOR pathway and simultaneously inhibited the expression of PD-L1 in gastric cancer cells, thus enhancing the antitumor immunity of T cells. This study provides a theoretical basis for applying gypenoside as a new therapeutic agent to enhance the efficacy of immunotherapy in gastric cancer.

## 1 Introduction

According to the latest global cancer statistics, gastric cancer (GC) is the fifth most common cancer (Sung et al., 2021; Siegel et al., 2022). Due to the lack of obvious symptoms of early gastric cancer, most patients are diagnosed with advanced GC. The survival period of patients with advanced GC is only 9–10 months, and the 5-year survival rate of patients with malignant advanced gastric cancer is still no more than 30% (Global Burden of Disease Cancer et al., 2022). Current treatments for GC include endoscopic resection, surgical resection, chemoradiotherapy, targeted therapy, and immunotherapy (Joshi and Badgwell, 2021). Major advances in immune checkpoint blockade have been made over the past 10 years, and immune checkpoint blockade has shown promising results in a spectrum of malignancies (Li et al., 2021). For GC, the pembrolizumab and nivolumab monoclonal antibodies for programmed cell death protein 1 (PD-1) have been approved (Bagchi et al., 2021). However, quite a few patients have a weak response to immunotherapy, resulting in poor treatment efficacy, and these agents are currently used only as second- and third-line regimens (Smyth et al., 2020; Joshi and Badgwell, 2021). Therefore, identifying new anti-gastric cancer immunotherapy drugs to improve the immune response is highly important.

PD-1 and programmed cell death ligand 1 (PD-L1) are second-generation immune checkpoint proteins (Sun et al., 2018). The interaction between PD-1 and PD-L1 negatively regulates the activation and function of T cells, eliminating T cells and leading to immunosuppression in cancer patients (Keir et al., 2008; Bagchi et al., 2021). Therefore, PD-1 or PD-L1 antibodies were used to block this pathway, thus enhancing antitumor immunity and inhibiting tumor growth. In the clinical treatment of GC, PD-1 or PD-L1 inhibitors have different benefits. The latest clinical studies have shown that pembrolizumab combined with trastuzumab and chemotherapy can significantly reduce tumor size. Compared to paclitaxel, pembrolizumab also increased the 24-month overall survival (OS) rate of PD-L1-positive patients, and there were fewer treatment-related adverse events. Although some patients benefit greatly, some patients do not strongly respond to the treatment due to immune escape, and the cost of immunotherapy is high (Janjigian et al., 2021; Fuchs et al., 2022). Therefore, exploring new drugs to avoid immune escape and increase the efficacy of immunotherapy for gastric cancer is particularly critical.

*Gynostemma pentaphyllum* (Thunb.) Makino, also known as Pentapanax leschenaultii, is a traditional Chinese medicine that belongs to the cucurbit family of the Gynostaphylla genus and is distributed in China, Japan, Korea, and Southeast Asia (He et al., 2019). The major active component is gypenoside, which has been used to treat atherosclerosis and hypoglycemia and has been reported to have protective effects on the liver and brain as well as exert immunomodulatory effects (Luo et al., 2022; Shu et al., 2022). Previous studies have demonstrated the antitumor activity of gypenoside in a variety of cancers, including lung cancer, pancreatic cancer, breast cancer, hepatocellular carcinoma, and colorectal cancer. (Li et al., 2016). The main antitumor mechanisms involve inducing cell apoptosis, inhibiting cell proliferation, and arresting the cell cycle (Ahmad et al., 2019). In terms of immunomodulatory effects, it has been reported that gypenoside inhibits inflammation to improve diabetic cardiomyopathy and protect retinal ganglion cells. However, the antitumor effect of gypenoside through immunomodulation has not been reported, and the role of gypenoside in the treatment of gastric cancer has not been determined.

In this study, we found that gypenoside induced the apoptosis of gastric cancer cells through the PI3K/AKT/mTOR pathway. Furthermore, network pharmacological analysis revealed that gypenoside could bind to STAT3 and reduce its phosphorylation. Thus, the transcription of PD-L1 was inhibited in gastric cancer cells, and the antitumor immunoregulatory effect of T cells was enhanced. These findings suggest that gypenoside may be used to enhance the efficacy of immunotherapy in gastric cancer.

## 2 Materials and methods

### 2.1 Materials

Gypenoside was purchased from Selleck.cn (cat. no. S5151). The fluorescent dye calcein-AM (CSA.148504-34-1) was purchased from Sigma‒Aldrich; DAPI (cat. no. C0121) and PI (cat. no. ST512) were purchased from Beyotime Biotechnology. The crystal violet dye was purchased from Beyotime Biotechnology (cat. no. C0121). A PE Annexin V Apoptosis Detection Kit with 7-AAD was purchased from BioLegend (cat. no. 640934). The antibodies used were obtained from the following places: Cleaved-caspase3 (cat. no. 9661S), p-AKT (Ser473) (cat. no. 4060S), p-AKT (Thr308) (cat. no. 13038S), AKT (cat. no. 4865S), p-S6 (S235/236) (cat. no. 4858S), and Bax (cat. No. 2772S) were purchased from Cell Signaling Technology; STAT3 (cat. No. T55292S), p-STAT3 (Tyr705) (cat. No. T56566S), mTOR (cat. No. M030653S), and p-mTOR (Ser2448) (cat. no. T56571S) were purchased from ABMART; Bcl-2 (cat. no. sc-7382), Bcl-xl (cat. no. sc-8392), and S6K (cat. no. sc-8418) were purchased from Santa Ctuz Biotechnology; β-actin was purchase from Sigma‒Aldrich; and PD-L1 (cat. no. 66248) and p-S6K (Thr389) (28735-1-AP) were obtained from Proteintech. The EasySep™ Mouse CD8^+^ T-Cell isolation kit was purchased from Stem Cell (cat.no. 79853A).

### 2.2 Cell culture

The human gastric cancer cell lines HGC-27 and SGC-7901 and the mouse gastric cancer cell line MCF were purchased from Wuhan Procell Life Technology Co., Ltd. HGC-27, SGC-7901, and MCF cells were cultured in RPMI 1640 medium supplemented with 10% fetal bovine serum and 1% penicillin streptomycin. The cells were cultured at 37°C and 5% CO_2_.

Primary mouse CD8^+^ T cells were obtained from total splenocytes isolated from 8-week-old BALB/c mice using a mouse CD8^+^ T-cell isolation kit according to the manufacturer’s protocol. Isolated T cells were activated on plates coated with an anti-mouse CD3 antibody (Invitrogen, cat. no. 16-0031-83, 1:1,000) and an anti-mouse CD28 antibody (Invitrogen, cat. no. 16-0281-83, 1:2000) plus complete RPMI medium containing IL-2 (50 ng mL^−1^), 10% fetal bovine serum, 4 mM L-glutamine, 1% penicillin/streptomycin, and 55 mM beta-mercaptoethanol for 3 days. The cells were cultured at 37°C and 5% CO_2_.

### 2.3 Cell counting Kit-8 (CCK-8) assay

HGC-27 and SGC-7901 cells (5 × 10^3^) were seeded in 96-well plates and incubated overnight. The next day, the sections were subjected to gypenoside (0, 30, 60, 90, 120, 150, or 180 μg/mL) for 24 or 48 h. Then, 10 μL of CCK8 solution was added to each well after treatment. The culture was continued for 2 h in the incubator, and the absorbance was measured at 450 nm using a Varioskan Flash instrument. Three independent experiments were performed for each dose.

### 2.4 Apoptosis assay by flow

A PE Annexin V Apoptosis Detection Kit with 7-AAD was used for the flow cytometry apoptosis assay. HGC-27 and SGC-7901 cells were placed in 6-well plates overnight and treated with different concentrations of gypenoside for 24 h. After treatment, the supernatant was collected, the cells were digested with trypsin, and the cells were collected and washed twice with cold Cell Staining Buffer (BioLegend). Then, the cells were resuspended in 100 µL of Annexin V binding buffer, and 2.5 µL of Annexin V-PE and 2.5 µL of 7-AAD were added. Afterward, the cells were gently vortexed and incubated for 15 min at room temperature in the dark. Finally, 200 μL of Annexin V binding buffer was added to each tube, and the proteins were analyzed via flow cytometry.

### 2.5 Calcein AM/PI staining

The cells were placed on coverslips in 24-well plates and incubated overnight. After treatment with different concentrations of gypenoside for 24 h, the cells were gently washed with PBS and stained with calcein AM/PI detection solution for 30 min at 37°C. After the incubation, the staining results were observed under a fluorescence microscope and photographed (calcein AM, green fluorescence; PI, red fluorescence).

### 2.6 Western blotting

HGC-27 and SGC-7901 cells were treated with gypenoside for 24 h and lysed by ultrasonification after the addition of lysis buffer with proteasome inhibitors. Next, the total protein concentration was determined via the BCA method. The extracted proteins were separated by 8%–12% SDS‒PAGE and transferred to polyvinylidene fluoride membranes. Afterward, the membranes were blocked with 5% skim milk (dissolved in TBST solution) powder at room temperature for 2 h. The specific primary antibodies were incubated at 4°C overnight. In this study, we used the following antibodies: anti-β-actin (diluted 1:20,000), anti-Bcl-2 (diluted 1:1,000), anti-Bcl-xl (diluted 1:1,000), anti-Bax (diluted 1:1,000), anti-cleaved caspase-3 (diluted 1:1,000), anti-AKT (diluted 1:1,000), anti-p-AKT ^ser473^ (diluted 1:1,000), anti-p-AKT ^ser308^ (diluted 1:1,000), anti-mTOR (diluted 1:1,000), anti-p-mTOR ^ser2448^ (diluted 1:1,000), anti-S6K (diluted 1:1,000), anti-p-S6 (d ^ser235/236^iluted 1:1,000), anti-p-S6K ^Thr389^ (diluted 1:1,000), anti-STAT3 (diluted 1:1,000), anti-p-STAT3 ^Tyr705^ (diluted 1:1,000), and anti-PD-L1 (diluted 1:1,000). On the second day, the membranes were washed with TBST solution and then incubated with anti-mouse or anti-rabbit horseradish peroxidase secondary antibodies at room temperature for 1 h. The membranes were subsequently washed with TBST, after which the hypersensitive chemiluminescence kit was added to the membranes to visualize the bands. Finally, the bands were detected with a gel imaging system and analyzed with Quantity One software.

### 2.7 Identification of the potential molecular targets of 10 selected gypenoside compounds in gastric cancer

The SwissTargetPrediction database (http://www.swisstargetprediction.ch/) was used to predict the potential action targets of 10 selected monomers, and the species was limited to humans. Repeated targets were removed, and 94 potential targets were obtained. The keyword “gastric cancer” was subsequently entered into the GeneCards database (https://www.genecards.org/) to retrieve 12,936 gastric cancer-related genes.

### 2.8 Protein–protein interaction network

The potential targets of gypenoside and gastric cancer were compared by the online website Venn Diagram (http://jvenn.toulouse.inra.fr/app/index.html), and 90 shared targets were obtained and displayed in a Venn diagram. Then, 90 genes were input into the STRING website (https://cn.string-db.org/), and the data with a confidence level higher than 0.7 were selected to finally construct the protein‒protein interaction network. The results were imported into Cytoscape 3.7.1 to construct the network diagram. The node size and color represent the degree of importance in this network.

### 2.9 Go and KEGG functional enrichment analysis

Gene Ontology (GO) and Kyoto Encyclopedia of Genes and Genomes (KEGG) enrichment analyses of the hub genes were performed by inputting the genes into the online analysis platform (DAVID, https://david.ncifcrf.gov).

### 2.10 Molecular docking analysis

The crystallized structures of PI3K, Akt, mTOR, and STAT3 and the cocrystallized structures of those proteins with their ligands were obtained from the Protein Databank (https://www.rcsb.org/). The obtained protein structures were further processed by PyMOL software to remove the solvent and other conjunctions. The 3D structures of the 10 selected saponins were drawn by using ChemDraw software. The hydrogenation of receptor proteins to achieve automatic charge distribution and the addition of charge to the ligand to achieve conformation restriction were all performed by AutoDockTools. The docking grid box was also constructed by AutoDockTools. Docking was performed by using QuickVina-W, and the optimal binding conformation was screened based on the Lamarckian genetic algorithm (Hassan et al., 2017). The binding energy of each molecule was calculated, and the core targets and molecules were visualized by using PyMOL.

### 2.11 PD-L1 immunofluorescent staining

Tumor cells were placed on coverslips in 24-well plates at 1 × 10^5^ cells per well and incubated overnight. After treatment with gypenoside for 24 h, the cells were dehydrated and fixed with cold ethanol. The cells were subsequently washed with cold PBS three times and blocked with 5% skim milk at room temperature for 30 min. Afterward, the cells were washed with PBS and incubated with an anti-PD-L1 primary antibody at 4°C overnight. The next day, the cells were washed with PBS and incubated with donkey anti-mouse IgG (H + L) secondary antibody (Alexa Fluor 488) for 1 h in the dark. Then, the cells were washed with PBS, stained with DAPI for 5 min in the dark, and sealed with an antifluorescence quenching agent. Finally, the fluorescence film was observed and photographed with a Leica confocal microscope.

### 2.12 *In vitro* coculture of mouse CD8^+^ T cells and tumor cells

Primary mouse CD8^+^ T cells were activated with an anti-mouse CD3 antibody (1:1,000 dilution) and an anti-mouse CD28 antibody (1:2,000 dilution) for 72 h. Tumor cells (5 × 10^3^) were placed in 24-well plates and incubated overnight. The tumor cells were treated with gypenoside for 24 h. Then, activated CD8^+^ T cells (5 × 10^4^ per well) were added to 24-well plates containing tumor cells for coculture. After another 48 h of coculture, the culture medium was removed, and the cells were gently washed with PBS. The cells were subsequently fixed with 75% ethanol for 15 min, gently washed with PBS three times, and stained with 1% crystal violet solution for 20 min. After staining, the dye was gently removed with PBS three times, and the cells were photographed with a microscope. Afterward, the cells were dissolved in 20% acetic acid. Finally, 100 µL of solution was added to a 96-well plate, and the absorbance was measured at 590 nm using a Varioskan Flash.

### 2.13 Animals and treatment

BALB/c mice (8 weeks old) were purchased from Huafukang Biotechnology Co., Ltd. (Beijing). MCF cells (3 × 10^6^ cells/mouse) were resuspended in serum-free medium and injected subcutaneously into the right leg of each mouse to construct the MCF subcutaneous transplanted tumor model. Mice were randomly classified into two groups (n = 5 per group). Ten days after tumor inoculation, the gypenoside group was intraperitoneally (i.p.) injected with gypenoside at a dose of 50 mg/kg/day. The tumor length and width were measured daily with a Vernier caliper and the tumor volume was calculated by the following formula: (volume = (width2 × length)/2). All of the animals were sacrificed after 10 days of drug exposure. Tumor tissues were examined by immunofluorescence and hematoxylin and eosin (H&E) staining.

### 2.14 Statistical analysis

The data were statistically analyzed with GraphPad Prism 8 software, and one-way ANOVA was used for comparisons between groups. *p* < 0.05 was considered to indicate statistical significance.

## 3 Results

### 3.1 The main active ingredients of gypenoside

**FIGURE 1:**
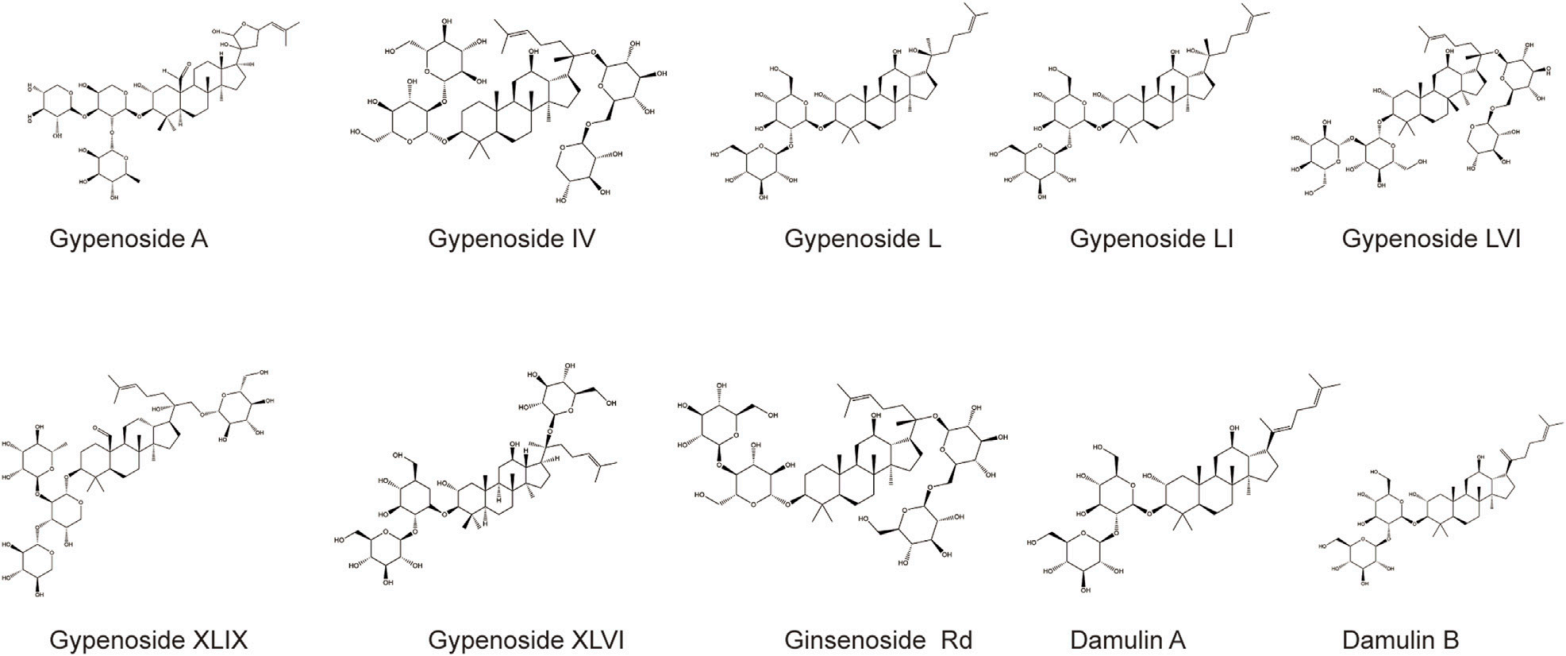
The molecular structures of the 10 compounds with the high gypenoside content.

Gypenoside was extracted from *G. pentaphyllum* (Thunb.) Makino, and the main extraction methods included water extraction, organic solvent extraction, ultrasonic extraction, enzyme extraction, and microwave extraction, as shown in Table 1. Among the extraction methods, microwave extraction has the shortest extraction time and the highest extraction efficiency. The efficiency of water extraction is low, and it is difficult to remove inorganic salts, proteins, and polysaccharides. The organic solvent extraction method involved the use of methanol, ethanol, n-butanol, chloroform, and other organic solvents. It is a relatively simple extraction technique that is widely used. The soluble materials can be removed, but the organic solvents are not easy to recover with this method. The ultrasonic extraction method is simple and the extraction process is not easily disrupted, but this method requires costly equipment, making mass production difficult. The enzyme extraction method involves the use of pectinase, hemicellulose, cellulase, and other complex enzymes. This approach has many advantages, such as rapid operation, high efficiency, short reaction time, and easy control. However, the conditions are strict and the operation is complicated, which makes it difficult to reproduce (Qiu et al., 2008; Zhang et al., 2014; Wang, 2018). At present, scientists have isolated and identified more than one hundred saponins from *G. pentaphyllum* (Thunb.) Makino (Su et al., 2021). Through a literature review and database comparison, we screened 10 compounds with high gypenoside content (Kao et al., 2008; Wu et al., 2014) (Table 2). In addition, the molecular structures of the 10 monomers were determined with ChemDraw (Figure 1).

**TABLE 1:** Extraction methods of gypenosides.

Extraction method	Reagent for extraction	Merit and demerit
Water extraction	Water	The cost is low, but the extration rate is low and it is difficult to remove inorganic salts, proteins and polysaccharides
Organic solvent extraction	Methanol, ethanol, butanol, chloroform, Petroleum ether,etc	A relatively simple extraction technique widely used. Soluble magazines can be removed, but it is not easy to recover
Ultrasonic extraction	Water	The operation is simple, the extraction rate is high, extract structure is not easy to be destroyed, but it requires high equipment and difficult to mass produce
Enzyme extraction	Pectinase, pectinase,etc	Fast, efficient, mild reation time and easy to control, but the conditions are strict and the operation is complicated, which makes it difficult to produce
Microwave extraction	Ethyl alcohol	Obviously shorten the extration time and increase the extrarion rate, it an extraction method worthy of application

**TABLE 2:** Compounds from *Gynostemma pentaphyllum* (Thunb.) Makino with high gypenoside content.

Molecule name	Molecular formula	Molecular weight
Gypenoside A	C_46_H_74_0_17_	899.1
Gypenoside IV	C_53_H_90_0_22_	1,079.3
Gypenoside LVI	C_53_H_90_0_23_	1,095.3
Gypenoside XLVI	C_49_H_82_0_19_	963.2
Ginsenoside Rd	C_48_H_82_0_18_	947.2
Gypenoside L	C_42_H_72_0_i4_	801
Gypenoside LI	C_42_H_72_0_14_	801
Gypenoside XLIX	C_52_H_86_0_21_	1,047.2
Damulin A	C_47_H_70_0_13_	783
Damulin B	C_47_H_70_0_13_	783

### 3.2 Gypenoside induces apoptosis in gastric cancer cells

**FIGURE 2:**
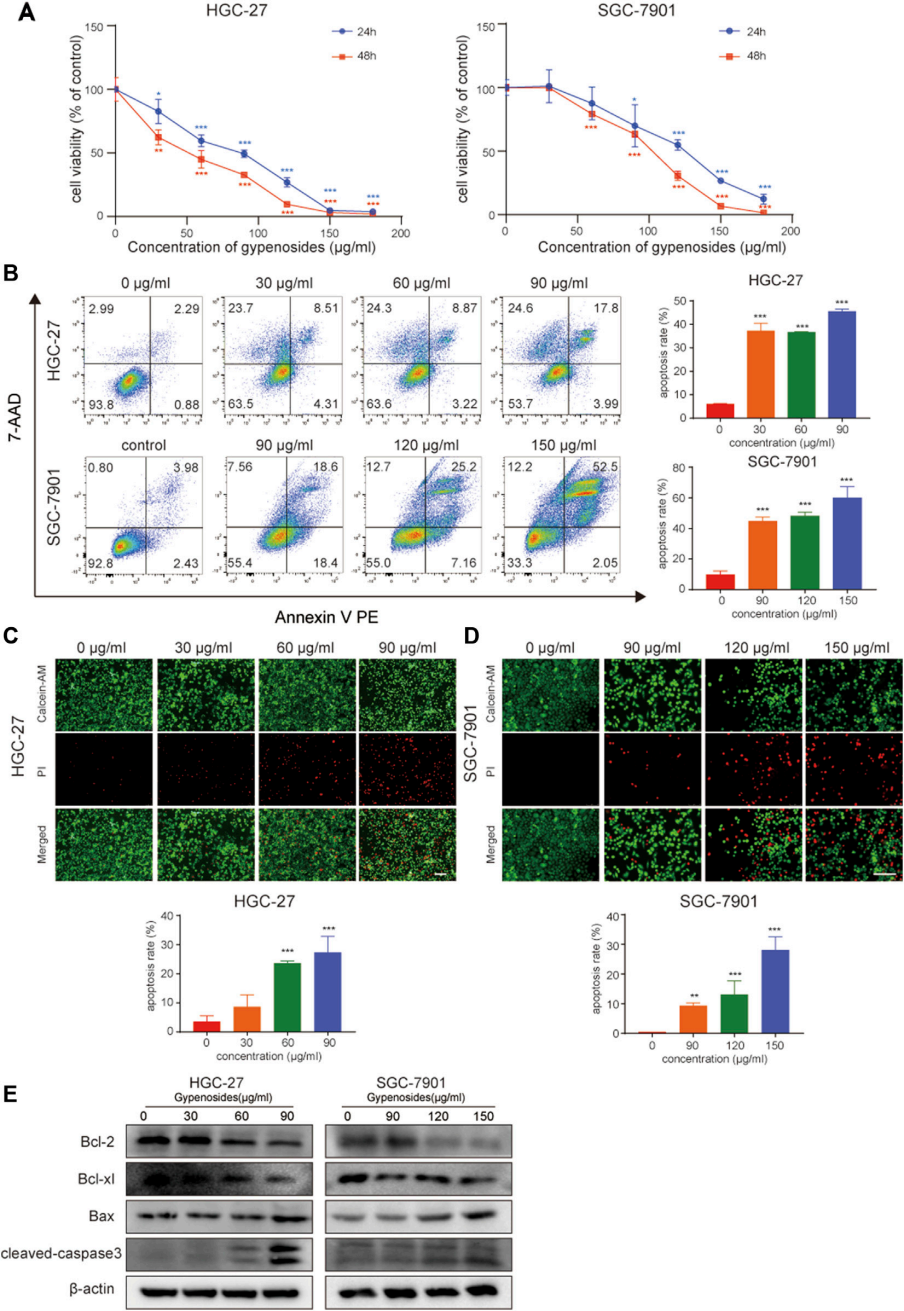
Gypenoside induces gastric cancer cell apoptosis. **(A)** HGC-27 and SCG-7901 cell viability was determined by an MTT assay. Cells were treated with different concentrations of gypenoside (0, 30, 60, 90, 120, 150, and 180 μg/mL) for 24 or 48 h. All of the data are expressed as mean ± SD from three independent experiments. ***p < 0.001 vs. the HGC-27 control group or SGC-7901 control group. **(B)** Apoptosis of HGC-27 and SGC-7901 cells was determined by flow cytometry. Cells were treated with different concentrations of gypenoside (0, 30, 60, or 90 μg/mL in HGC-27 cells or 0, 90, 120, or 150 μg/mL in SGC-7901 cells) for 24 h. All of the data are expressed as mean ± SD, from three independent experiments. ***p < 0.001 vs. the control group. **(C,D)** Apoptosis of HGC-27 and SGC-7901 cells was detected by calcein AM/PI staining. Cells were treated with different concentrations of gypenoside (0, 30, 60, or 90 μg/mL in HGC-27 cells or 0, 90, 120, or 150 μg/mL in SGC-7901 cells) for 24 h. Green indicates calcein AM and red indicates PI. Scale bar, 150 μm ***p < 0.001 vs. the control group. **(E)** The expression levels of apoptosis-related proteins Bcl-2, Bcl-xl, Bax, and cleaved caspase-3 in HGC-27 and SGC-7901 cells after treatment with gypenoside were detected via Western blotting.

A large number of studies have reported that gypenoside can induce morphological changes and apoptosis and inhibit the proliferation of various cancer cells, such as lung cancer (Li et al., 2021; Qi et al., 2021), breast cancer (Zu et al., 2021; Tan et al., 2022), and kidney cancer cells (Liu et al., 2021). However, the effect of gypenoside on gastric cancer is unclear. We first evaluated the effect of gypenoside on the cytotoxicity of gastric cancer cells. The CCK-8 assay showed that gypenoside inhibited the growth of HGC-27 and SGC-7901 cells in a concentration-dependent and time-dependent manner (Figure 2A). The survival rates of cancer cells were less than 50% when the concentration was 50 μg/mL for HGC-27 cells and 100 μg/mL for SGC-7901 cells. To further investigate the cytotoxic effect on gastric cancer cells, flow cytometry was used to detect the apoptosis levels of HGC-27 and SGC-7901 cells after 24 h of gypenoside treatment. Gypenoside treatment promoted the apoptosis of gastric cancer cells, and the percentage of apoptotic cells increased with increasing drug concentration (Figure 2B). Moreover, according to the results of the calcein AM/PI staining assay, the number of dead cells in the gypenoside treatment groups was much higher than that in the DMSO treatment group, and the number of dead cells increased with increasing concentration (Figure 2C). Furthermore, we detected the expression of the apoptosis-related proteins Bcl-2, Bcl-xl, Bax, and cleaved caspase 3. The expression of Bcl-2 and Bcl-xl was more downregulated, and the expression of Bax and cleaved caspase 3 was more upregulated in the treatment group compared to the control group (Figure 2D). These results indicated that gypenoside could induce the apoptosis of gastric cancer cells.

### 3.3 Common targets of gypenoside and gastric cancer

**FIGURE 3:**
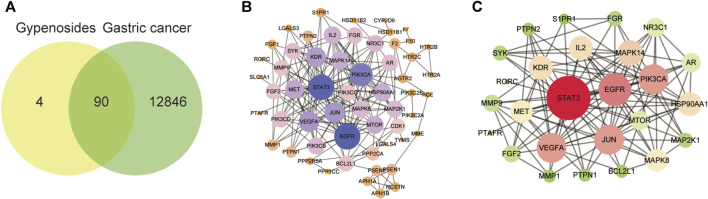
Gastric cancer-related genes that may be targeted by gypenoside. **(A)** Venn diagram revealing the genes involved in the interaction between gypenoside and gastric cancer. **(B)** Protein‒protein interaction (PPI) network of gastric cancer-associated proteins. Larger node sizes and darker colors indicate greater degrees of association. **(C)** The main network in the PPI network. Larger node sizes and darker red colors indicate a higher degree and green colors indicate a lower degree of association.

To explore the mechanism by which gypenoside induces the apoptosis of gastric cancer cells, we predicted the potential targets of 10 screened gypenoside-containing compounds through the Swiss database and removed the repeated targets via UniProt. We obtained 94 potential targets of the 10 compounds. Moreover, we used gastric cancer as a key word in the GeneCards database to obtain 12,936 gastric cancer-related targets. Subsequently, a total of 90 shared genes were identified via Venn analysis (Figure 3A). Next, the 90 shared genes were loaded into STRING software to construct the protein–protein network diagram (Figure 3B). We screened the major network sets according to the degree score (Figure 3C). The key genes are shown in Figure 3C. The top 10 genes according to degree of change were STAT3, EGFR, PI3KCA, VEGFA, JUN, MAPK14, IL-2, KDR, HSP90AA1, and MET. Therefore, we speculated that these 10 key genes might be involved in the mechanism through which gypenoside inhibits gastric cancer.

### 3.4 Gypenoside inhibits the PI3K-AKT-mTOR pathway in gastric cancer cells

**FIGURE 4:**
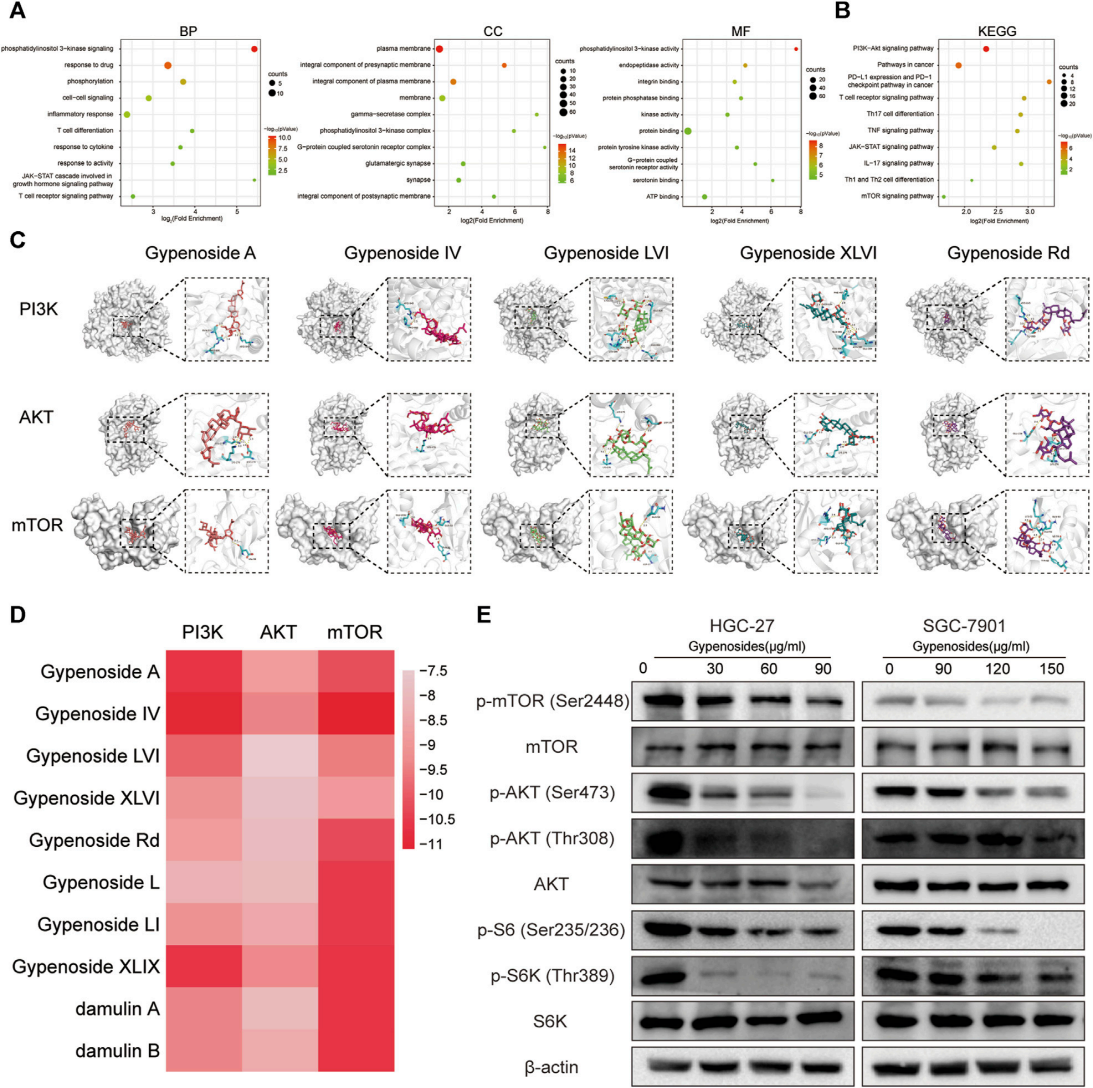
Gypenoside inhibits the PI3K-AKT-mTOR pathway in gastric cancer cells **(A)** Gene Ontology (GO) functional annotation of potential targets of gypenoside. Biological process (BP), cellular component (CC), and molecular function (MF) categories were analyzed. **(B)** Kyoto Encyclopedia of Genes and Genomes (KEGG) enrichment analysis of potential signaling pathways associated with gypenoside treatment. **(C)** Docking complex of three target proteins (PI3K, AKT, and mTOR) and five gypenoside components (gypenoside A, gypenoside IV, gypenoside LVI, gypenoside XLVI, and gypenoside Rd.). **(D)** Heatmap showing the docking scores of PI3K, AKT, and mTOR in combination with the other ten gypenoside components. **(E)** Protein expression of p-mTOR (Ser2448), mTOR, p-AKT (Ser473), p-AKT (Thr308), AKT, p-S6 (Ser235/236), p-S6K (Thr308), and S6K in HGC-27 and SGC-7901 cells after treatment with gypenoside for 24 h.

We investigated the specific molecular mechanism by which gypenoside induces apoptosis in gastric cancer cells. First, we inputted the 90 shared genes into the DAVID website for GO and KEGG enrichment analysis. According to the GO enrichment analysis, 363 terms were related to the biological process (BP), 69 terms were related to the cellular component (CC), and 76 terms were related to the molecular function (MF). We have listed the changes in the top 10 BPs, MFs, and CCs in a bubble diagram (Figure 4A). Subsequently, KEGG enrichment analysis revealed that the PI3K-Akt signaling pathway was enriched and involved 18 potential target genes. We identified 10 enrichment pathways, as shown in Figure 4B. Among the highly enriched genes, we found that the PI3K-AKT-mTOR signaling pathway is closely related to the growth of various tumor cells (Alzahrani, 2019; Yang et al., 2019). Moreover, we used molecular docking to verify whether gastric cancer cell apoptosis occurs through the PI3K-AKT-mTOR pathway. We evaluated the binding ability of the 10 monomers with the highest levels of PI3K, AKT, and mTOR. The results of the cluster analysis of the docking scores are shown in Figure 4D. Figure 4C shows the docking results for the five compounds with the highest concentrations. The molecular docking results revealed that the gypenoside strongly binds to PI3K, AKT, and mTOR. Furthermore, we detected the protein levels of members in the PI3K-AKT-mTOR pathway. The expression of p-mTOR (Ser2448), p-AKT (Ser473), p-AKT (Thr308), p-S6 (Ser235/236), and p-S6K (Thr389) was significantly downregulated after treatment with gypenoside in HGC-27 and SGC-7901 cells (Figure 4E). In conclusion, the PI3K-AKT-mTOR pathway is a key pathway through which gypenoside induces the apoptosis of gastric cancer cells.

### 3.5 Gypenoside is closely related to immunity in gastric cancer cells

The gene network analysis shown in Figure 3B revealed that the shared genes, including STAT3 and IL-2, are strongly related to immune regulation. KEGG enrichment analysis, shown in Figure 4B, also revealed a strong correlation with immune effector processes. For example, PD-L1 expression and the PD-1 checkpoint pathway are involved in cancer, while the T-cell receptor signaling pathway, Th17 cell differentiation, the TNF signaling pathway, the IL-17 signaling pathway, and Th1 Th2 are involved in cell differentiation. GO analysis, as shown in Figure 4A, suggested that the shared genes are enriched in the Jak-STAT and T-cell receptor signaling pathways related to immune regulation. In conclusion, gypenoside is speculated to inhibit the growth of gastric cancer cells by enhancing the antitumor immunity of T cells, possibly through modulating the expression of PD-L1 on the surface of gastric cancer cells.

### 3.6 Gypenoside inhibits the phosphorylation of STAT3 and downregulates the expression of PD-L1

**FIGURE 5:**
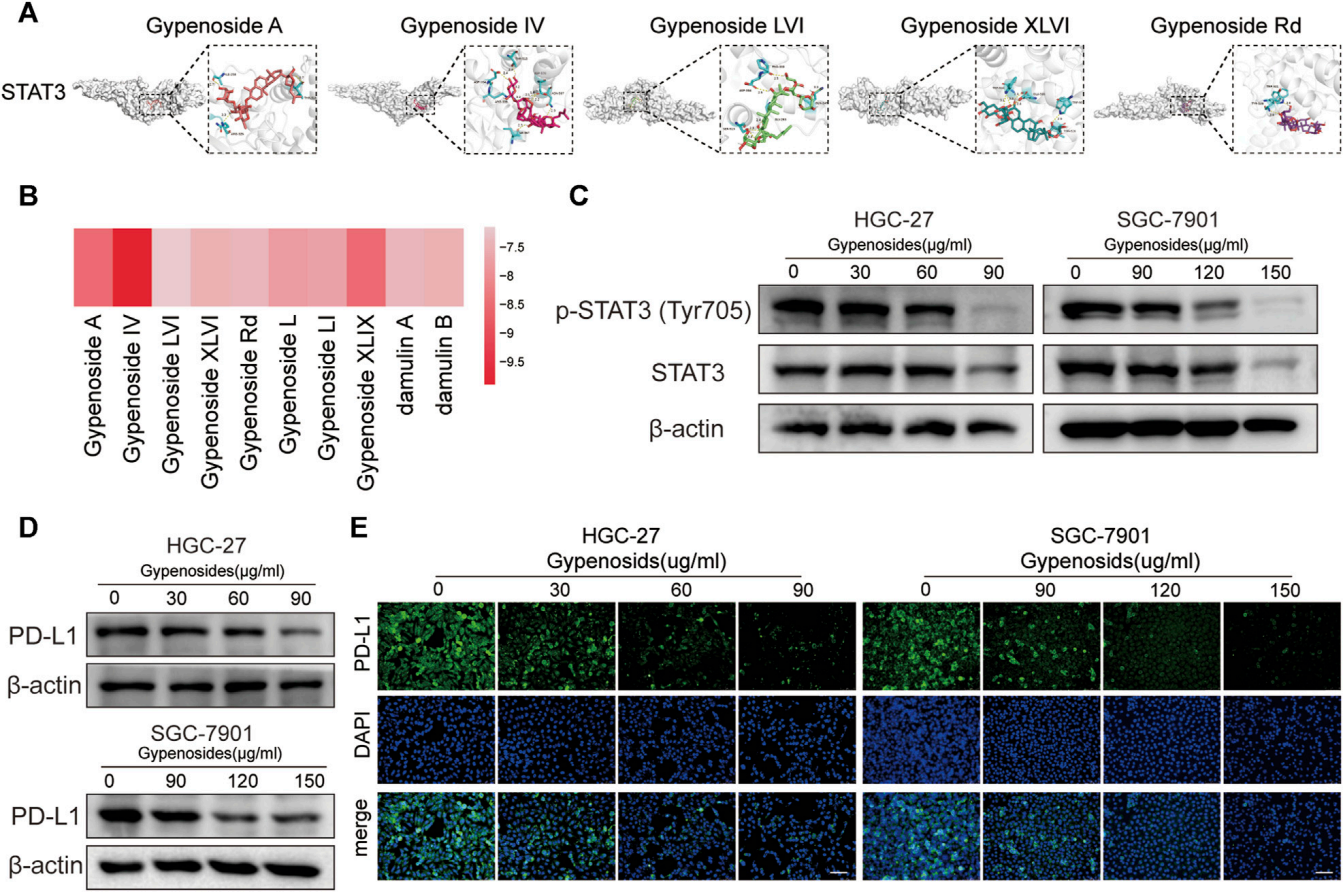
Gypenoside inhibits the phosphorylation of STAT3 and downregulates the expression of PD-L1. **(A)** Docking complex of STAT3 and five gypenoside components (gypenoside A, gypenoside IV, gypenoside LVI, gypenoside XLVI, and gypenoside Rd.). **(B)** Heatmap showing the docking scores of STAT3 in combination with the other ten gypenoside components. **(C)** The protein expression levels of P-STAT3 (Tyr705) and STAT3 in HGC-27 and SGC-7901 cells after treatment with gypenoside were detected by Western blotting. **(D)** The protein expression levels of PD-L1 in HGC-27 and SGC-7901 cells after treatment with gypenoside. **(E)** HGC-27 cells were exposed to 0, 30, 60, or 90 μg/mL, and SGC-7901 cells were exposed to 0, 90, 120, or 150 μg/mL for 24 h. The PD-L1 (green) in the cells was labeled, and the nuclei were stained with DAPI (blue) prior to imaging via confocal microscopy. Scale bar, 75 μm.

It has been reported in the literature that p-STAT3, the phosphorylated product after STAT3 activation, can directly bind to the PD-L1 promoter region, which promotes the transcription of PD-L1 and affects its expression (Herrmann et al., 2010; Koh et al., 2016; Xie et al., 2021). The results of our network pharmacological analysis showed that gastric cancer cells treated with gypenoside were significantly correlated with STAT3 (Figures 3B, C and Figures 4A, B). To verify the possibility of gypenoside acting on STAT3 and PD-L1, we conducted molecular docking studies on 10 monomers with high gypenoside content and STAT3. The results suggested that all 10 monomers could bind to STAT3, and the results of the molecular docking of the top 5 high-content monomers are shown in Figure 5A. The docking scores were satisfactory, and the docking scores of all the monomers are displayed in the heatmap in Figure 5B. Gypenoside IV was the monomer with the highest degree of interaction. Second, we detected the protein expression of STAT3 and p-STAT3 in gastric cancer cells after gypenoside treatment. The results showed that the expression level of p-STAT3 (Tyr705) gradually decreased with increasing drug concentration, while the expression of STAT3 did not change at low concentrations (Figure 5C). With high concentrations of gypenoside, the expression level of STAT3 decreased, which is consistent with the findings of Yanshuang Qi et al., who reported that gypenoside can also inhibit the expression of STAT3 (Qi et al., 2021). p-STAT3 can reportedly promote the transcription of PD-L1 (Koh et al., 2016). However, if the phosphorylation of STAT is reduced, p-STAT does not enter the nucleus, thus inhibiting the transcription of PD-L1 and downregulating the expression of PD-L1, which further affects immune regulation. We further detected changes in PD-L1 protein levels in HGC-27 and SGC-7901 cells and found that PD-L1 protein levels were significantly decreased after gypenoside treatment in gastric cancer cells (Figure 4D). Similarly, the immunofluorescence staining results revealed downregulated PD-L1 expression in HGC-27 and SGC-7901 cells after treatment (Figure 4E). In summary, gypenoside reduced the phosphorylation of STAT3 and inhibit the expression of PD-L1 in gastric cancer cells.

### 3.7 Gypenoside enhances T-cell killing to induce apoptosis in gastric cancer cells

**FIGURE 6:**
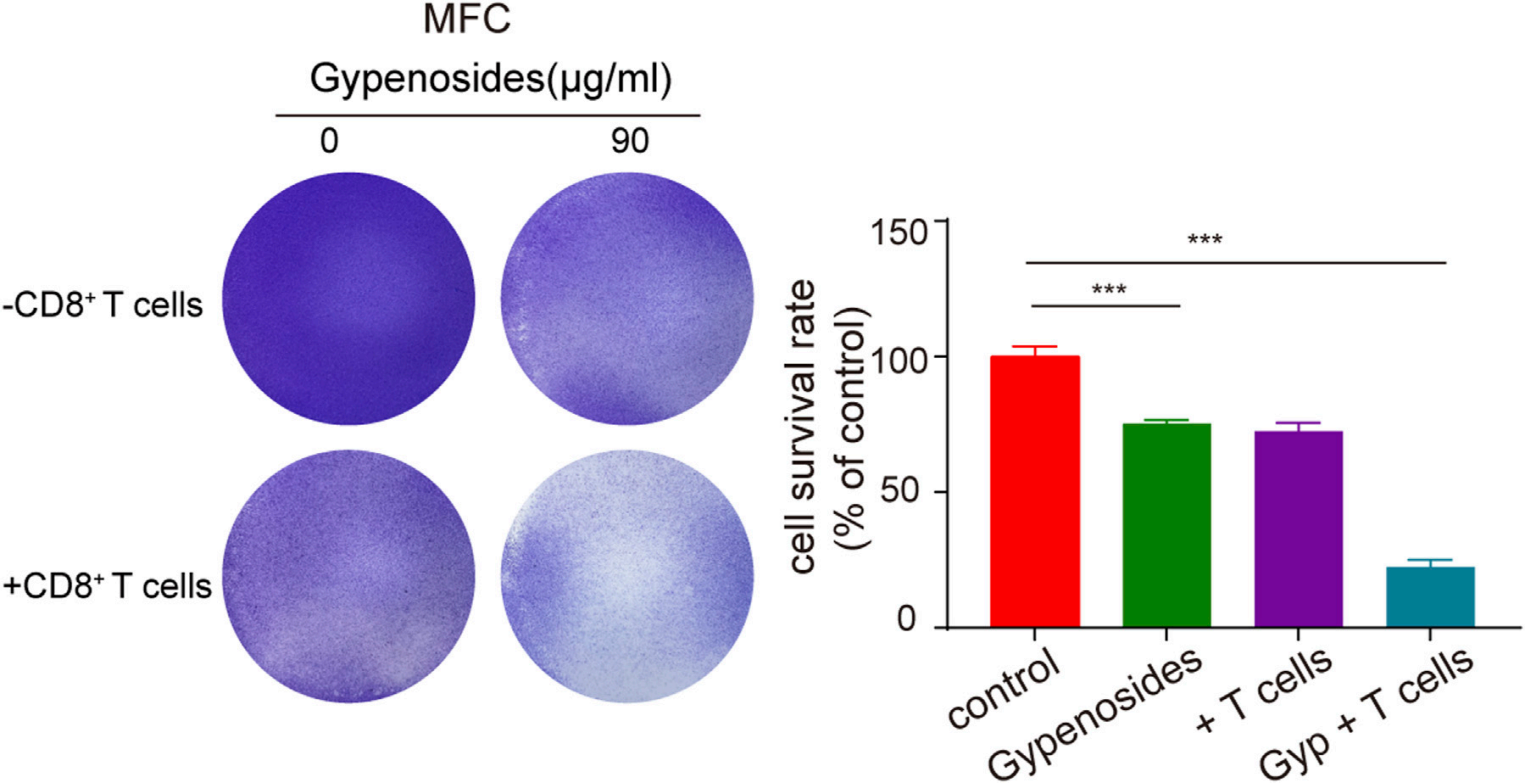
Gypenoside enhances the killing of gastric cancer cells by mouse CD8^+^ T cells. Mouse gastric cancer cells were treated with gypenoside for 24 h in 24-well plates and cocultured with stimulated mouse CD8^+^ T cells for 72 h. At the indicated time points, crystal violet staining was performed, and the cells were photographed with a microscope. The bar chart shows the ratio of the absorbance at 590 nm relative to that of the control group. ***p < 0.001 vs. the control group.

Many studies have shown that the combination of PD-L1 on the surface of tumor cells and PD-1 on the surface of T cells initiates the programmed death of T cells, enabling tumor cells to achieve “immune escape”, and that PD-1/PD-L1 immune checkpoint inhibitors can restore the antitumor activity of T cells by blocking the binding of PD-1 to PD-L1 (Doroshow et al., 2021; Yamaguchi et al., 2022). Once the antitumor immune cycle is established, lasting antitumor effects can be produced. To enhance the antitumor activity of T cells, one can block the binding of PD-1 to PD-L1 through immune checkpoint inhibitors or downregulate the expression of PD-L1 or PD-1. The downregulation of PD-L1 expression in gastric cancer cells can activate T-cell immunity and enhance aggression toward tumor cells (Dammeijer et al., 2020; Topalian et al., 2020). We cocultured primary mouse CD8^+^ T cells with mouse gastric cancer cells and found that the apoptotic effect was significantly greater in the T-cell coculture plus gypenoside group than in the coculture without administration group and in the nonculture group (Figure 6). These results indicate that gypenoside can enhance the antitumor immunity of T cells and enhance the aggression of T cells against gastric cancer cells by inhibiting the expression of PD-L1 in gastric cancer cells.

### 3.8 Gypenoside inhibits tumor growth, induces apoptosis, and downregulates the expression of PD-L1 in an MCF subcutaneous transplanted tumor model

**FIGURE 7:**
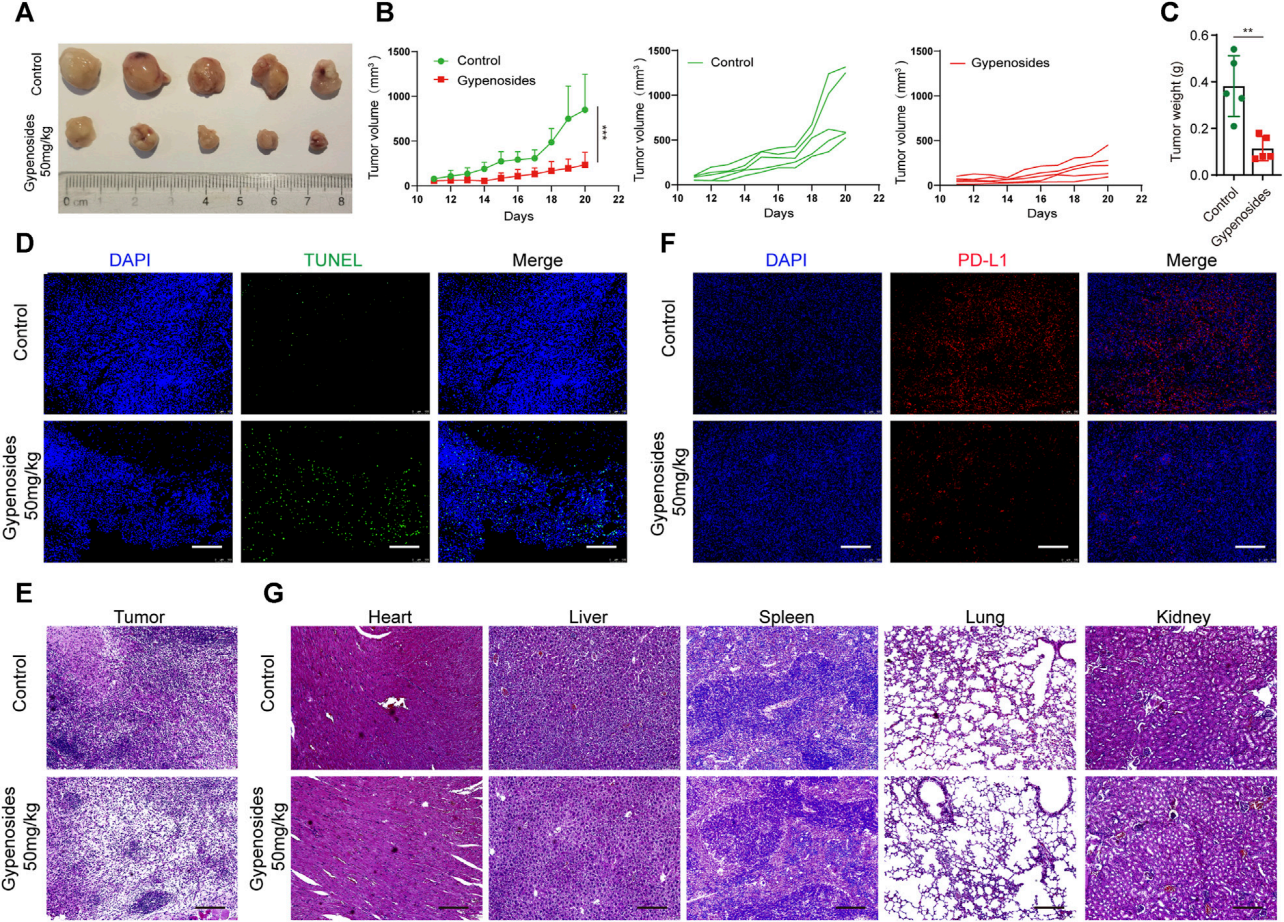
Gypenoside inhibited tumor growth, induced apoptosis, and downregulated the expression of PD-L1 in an MCF subcutaneous transplanted tumor model. **(A)** Representative images of each tumor in each group. **(B)** Tumor growth curves for control and gypenoside-treated mice (*n* = 5). **(C)** Tumor weights of mice in the control and gypenoside treatment groups. **(D)** Tumor tissues were fixed and subjected to immunofluorescence staining for TUNEL. Scale bar, 200 μm. **(E)** Representative H&E-stained tumor sections from control and gypenoside-treated mice. Scale bars: 200 μm. **(F)** Tumor tissues were stained for PD-L1, the PD-L1 level (red) was measured, and nuclei were stained with DAPI (blue) prior to imaging via confocal microscopy. Scale bar, 200 μm. **(G)** Representative H&E-stained heart, liver, spleen, lung and kidney Sections from control and gypenoside-treated mice. Scale bars, 200 μm. **p < 0.01, ***p < 0.001 vs. the control group.

To determine the antitumor activity of gypenoside *in vivo*, we constructed a gastric cancer subcutaneous transplanted tumor model using BALB/c mice (Figures 7A–C). Our results revealed that gypenoside effectively inhibited tumor growth. The tumor volume and tumor weight in the gypenoside administration group were significantly lower than those in the control group. Furthermore, immunofluorescence staining of tumor tissues via TUNEL and immunohistochemical staining via H&E showed that gypenoside promoted tumor cell apoptosis (Figures 7D, E). Additionally, heart, liver, spleen, lung, and kidney samples were excised, sectioned, and analyzed via hematoxylin and eosin (H&E) staining. Our results showed that there were no morphological differences between the gypenoside-treated and control groups (Figure 7G). To determine whether the expression of PD-L1 was downregulated after gypenoside administration, immunofluorescence staining was performed on tumor tissue, and the results revealed that the expression of PD-L1 was significantly downregulated in the gypenoside-treated group (Figure 7F). These results suggest that gypenoside inhibits tumor growth and induces cell apoptosis in MCF subcutaneous transplanted tumor model mice in association with downregulated PD-L1 expression in tumor cells.

## 4 Discussion

In this study, we confirmed that gypeoside induces the apoptosis of gastric cancer cells, including HGC-27 and SGC-7901 cells, by inhibiting the PI3K-AKT-mTOR pathway. In addition, we also found that gypeoside can enhance the antitumor immunity of T cells by inhibiting the expression of PD-L1 in gastric cancer cells. Our study provides an experimental basis for the development of gypenoside as a new candidate antitumor immune drug for the treatment of gastric cancer.

The induction of tumor cell apoptosis is an effective strategy for antitumor therapy. Therefore, it is important to examine the effect and mechanism of antitumor drugs that induce tumor cell apoptosis. In recent years, many studies have reported the antitumor effect of gypenoside. For instance, gypenoside induces apoptosis in A549 cells by inhibiting NF-κB signaling and enhancing the expression level of caspase-3 and the Bax/Bcl-2 ratio (Xiaoli Li et al., 2021) (Li et al., 2021). Furthermore, gypenoside induces cell apoptosis by increasing intracellular oxidative stress levels in human esophageal cancer Eca-109 and colon cancer SW620 cells (Yan H et al., 2014) (Yan et al., 2014). Gypenoside inhibits colorectal cancer growth and metastasis by activating the p53 pathway and increasing oxidative stress-mediated DNA damage (Lulu Kong et al., 2015) (Kong et al., 2015). Moreover, gypenoside reportedly promoted cell apoptosis via the PI3K/AKT/mTOR signaling pathway in renal cell carcinoma and bladder cancer cells (Liu et al., 2021; Li et al., 2022). However, there are no reports on the role of gypenoside in gastric cancer cells. In our study, gypenoside treatment effectively reduced the viability of gastric cancer cells, including HGC-27 and SGC-7901 cells, as determined by a CCK-8 assay. Subsequently, flow cytometry, calcein AM/PI staining, and apoptosis-related protein detection revealed that gypenoside treatment promoted the apoptosis of HGC-27 and SGC-7901 cells.

The PI3K/AKT/mTOR signaling pathway plays an important role in the progression of various cancers and plays an extremely important role in cell growth, survival, proliferation, angiogenesis, autophagy, and other processes (Janku et al., 2018). PI3K is a dimer composed of two subunits: the catalytic subunit p110 and the regulatory subunit p85. When PI3K binds to different receptors, it can change the cell state by regulating the activity of downstream proteins. For example, when PI3K binds to growth factor receptors such as EGFR, it can be activated through protein structural changes in AKT, which activate or inhibit the activity of downstream substrates such as proliferation-associated proteins and apoptosis-related proteins through AKT phosphorylation, thereby regulating cell proliferation, migration, and apoptosis (Fruman et al., 2017; Vasan and Cantley, 2022). Moreover, the PI3K/AKT/mTOR pathway plays a key role in promoting cell survival by inhibiting B-cell lymphoma-1 (Bcl-2), Bax, and caspase-3 and upregulating antiapoptotic proteins such as NF-κB (Fruman and Rommel, 2014; LoRusso, 2016). Moreover, through a comprehensive network pharmacology analysis of the potential sites of action of gypenosides in gastric cancer, we found that the PI3K/AKT/mTOR pathway plays a crucial role in the apoptosis of gastric cancer cells. Subsequently, we used molecular docking and Western blotting to confirm that gypenosides inhibited the PI3K/AKT/mTOR signaling pathway to induce apoptosis in gastric cancer cells.

In recent years, immunotherapy has made a historic breakthrough in cancer therapy. Antitumor therapy involving immune checkpoints has attracted widespread attention. Immunosuppressive molecules on the surface of T cells or NK cells bind to their corresponding ligands to induce nonreactivity of T cells or NK cells and generate immunosuppression, while immune checkpoint blockade therapy enhances the aggression of the host immune system toward tumor cells by inhibiting the binding of immunosuppressive receptors and ligands, which is crucial for activating the antitumor immunity of T cells. How to extend clinical benefits to the majority of cancer patients and explore new options for enhanced immunotherapy has been a focus in this area. The efficacy of immunotherapy can be improved by using immune checkpoint inhibitors (Sun et al., 2018; Doroshow et al., 2021; Myers and Miller, 2021). Currently, the FDA has approved three immune checkpoint inhibitors: CTLA-4, PD-1/PD-L1, and LAG3. Although immune checkpoint blockade therapy is effective, it is expensive, and individual responses vary. Moreover, the antitumor immune response can be activated by inhibiting the expression of immunosuppressive molecules (CTLA-4/PD-1/CTLA4, *etc.*) or their ligands (PD-L1, *etc.*) (Pardoll, 2012). In our study, the common targets of gypenoside and gastric cancer were enriched in the biological process of the immune response, as well as in PD-L1 expression and the PD-1 checkpoint pathway. Western blot analysis revealed that gypenoside could downregulate the expression of PD-L1 in gastric cancer cells, and immunofluorescence staining of PD-L1 confirmed this finding in gastric cancer cells after drug treatment. According to the results of the GO enrichment analysis, the common targets of gypenoside and gastric cancer were enriched in the Jak-STAT pathway. Several scholars have reported that the phosphorylation of STAT3 decreases, which can inhibit the entry of activated p-STAT3 into the nucleus, and that p-STAT3 can bind to the promoter of PD-L1 to promote its transcription. Therefore, we observed the changes in the protein levels of STAT3 and p-STAT3 and found that the level of STAT3 was unchanged and that the level of p-STAT3 was decreased. In conclusion, gypenoside can downregulate the expression of PD-L1 by inhibiting the translocation of STAT3 phosphate into gastric cancer cells. However, whether downregulated PD-L1 expression in gastric cancer cells results in immune activation was unclear. By coculturing gastric cancer cells treated with drugs and T cells, we showed that downregulated PD-L1 expression in gastric cancer cells activated antitumor T cells and promoted the death of gastric cancer cells. Collectively, these results showed that gypenoside can enhance the antitumor effects of T cells by reducing PD-L1 expression levels in gastric cancer cells.

In summary, gypenoside induced the apoptosis of HGC-27 and SGC-7901 gastric cancer cells by inhibiting the PI3K/AKT/mTOR pathway and enhancing the antitumor effects of T cells by inhibiting the expression of PD-L1. Our findings provide a mechanistic basis for the application of gypenoside in gastric cancer immunotherapy.

## Data Availability

The original contributions presented in the study are included in the article/Supplementary Material, further inquiries can be directed to the corresponding authors.
