# Supplementary figures and images for: Gypenoside induces apoptosis by inhibiting the PI3K/AKT/mTOR pathway and enhances T-cell antitumor immunity by inhibiting PD-L1 in gastric cancer
Source: Front Pharmacol. 2024 Feb 28;15:1243353. doi: 10.3389/fphar.2024.1243353 (PMC10933075; doi:10.3389/fphar.2024.1243353)

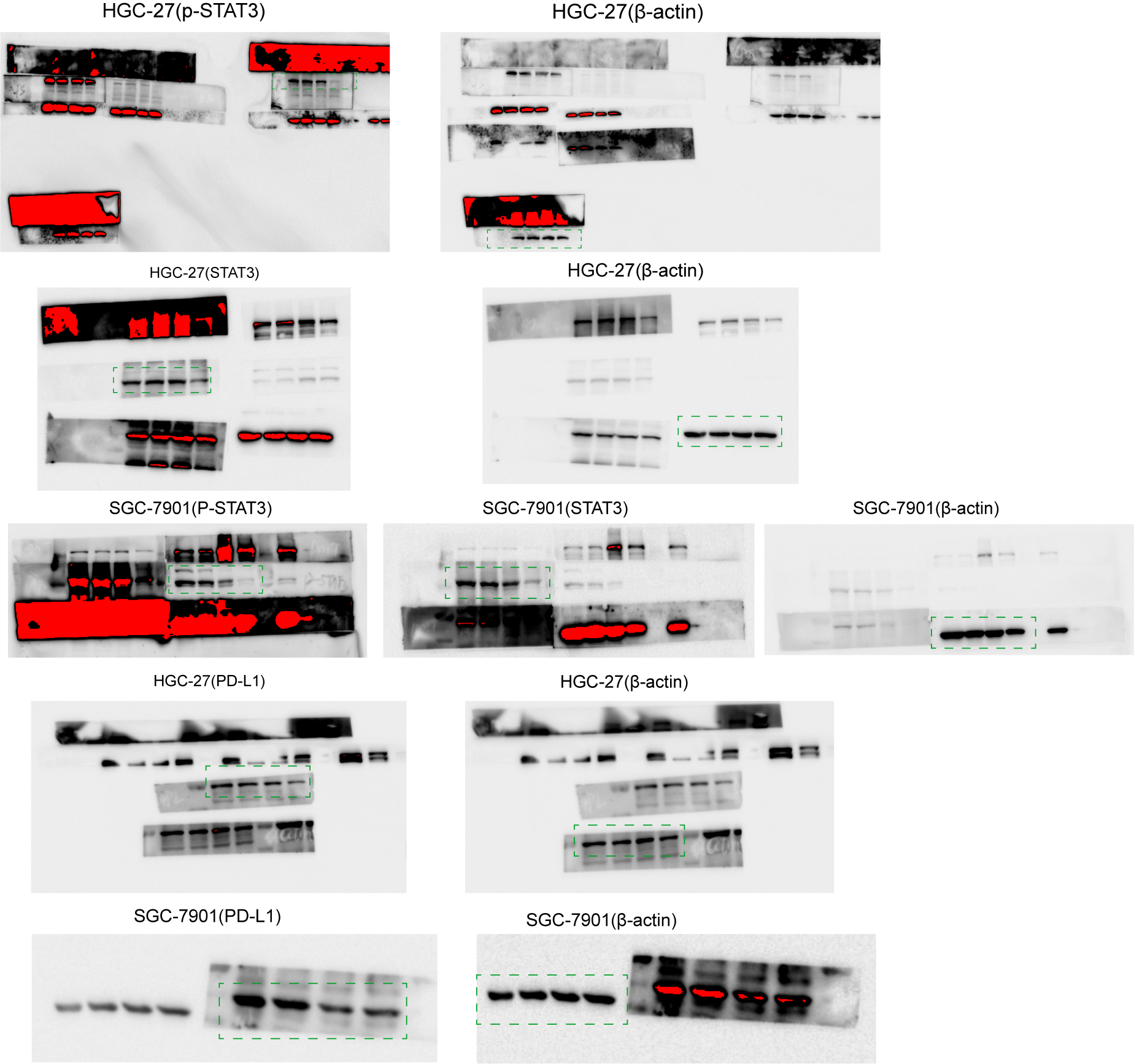

Supplement: Supplementary file 3 [file Image6.TIF]

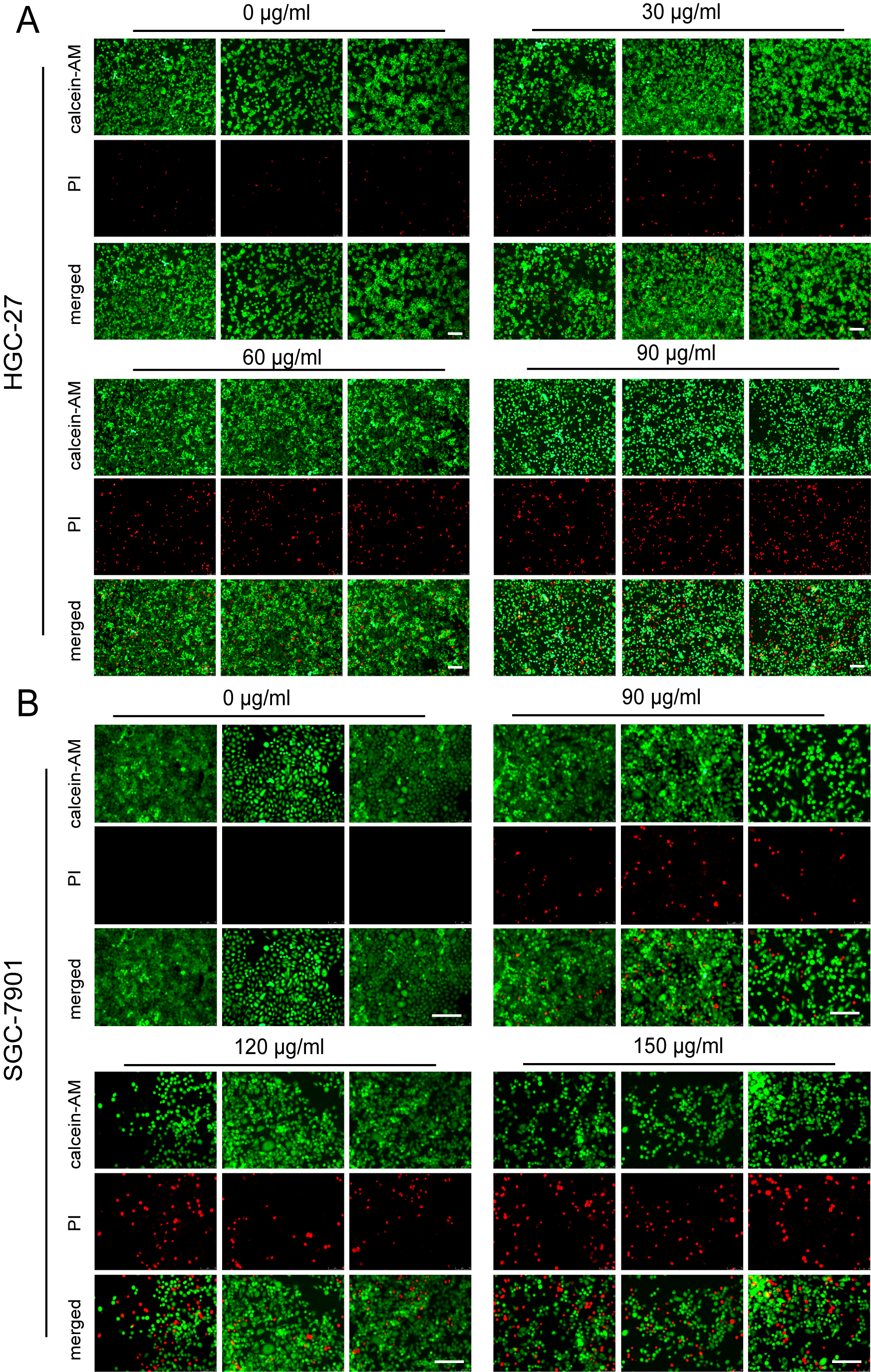

Supplement: Supplementary file 5 [file Image3.TIF]

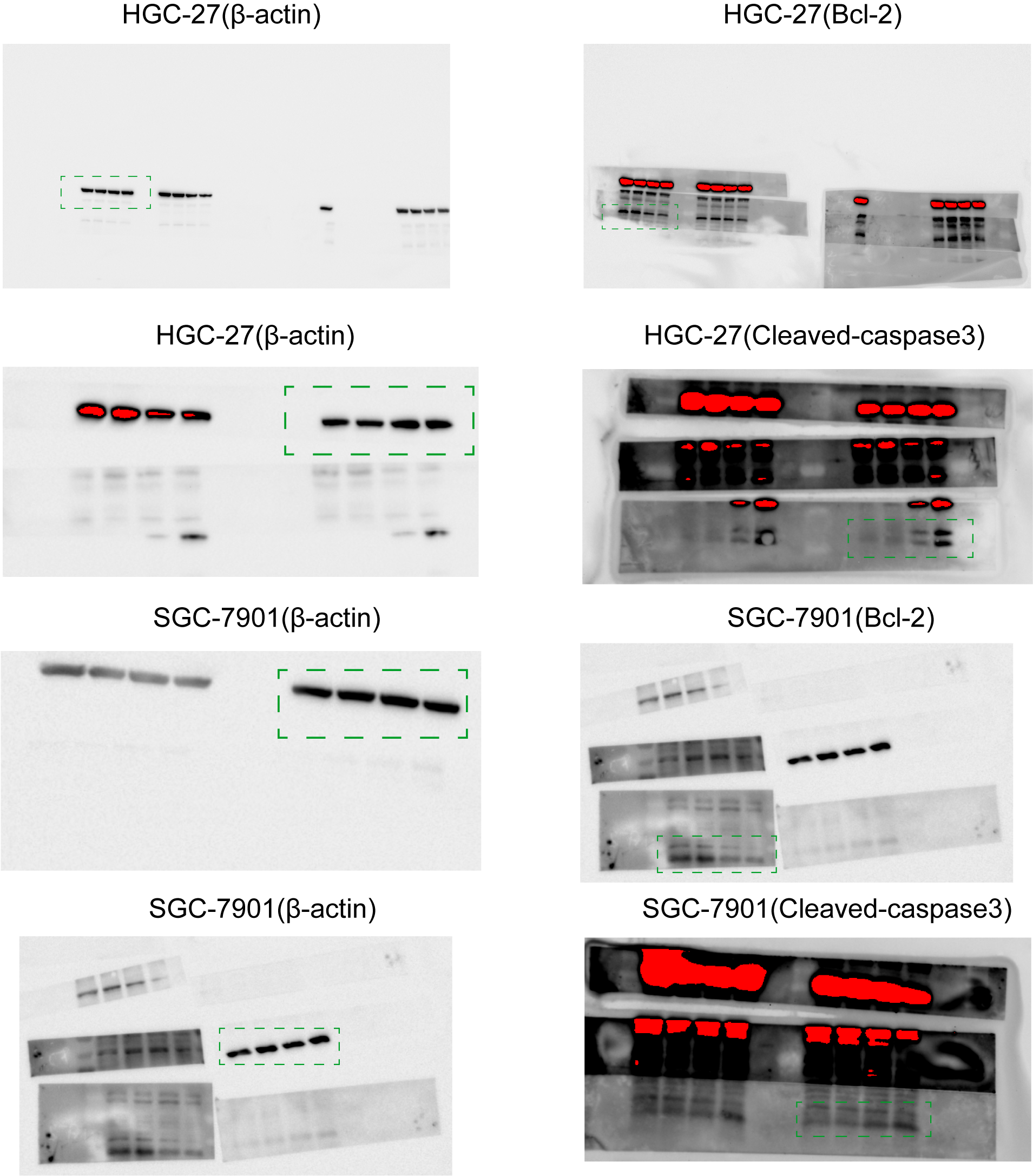

Supplement: Supplementary file 6 [file Image4.TIF]

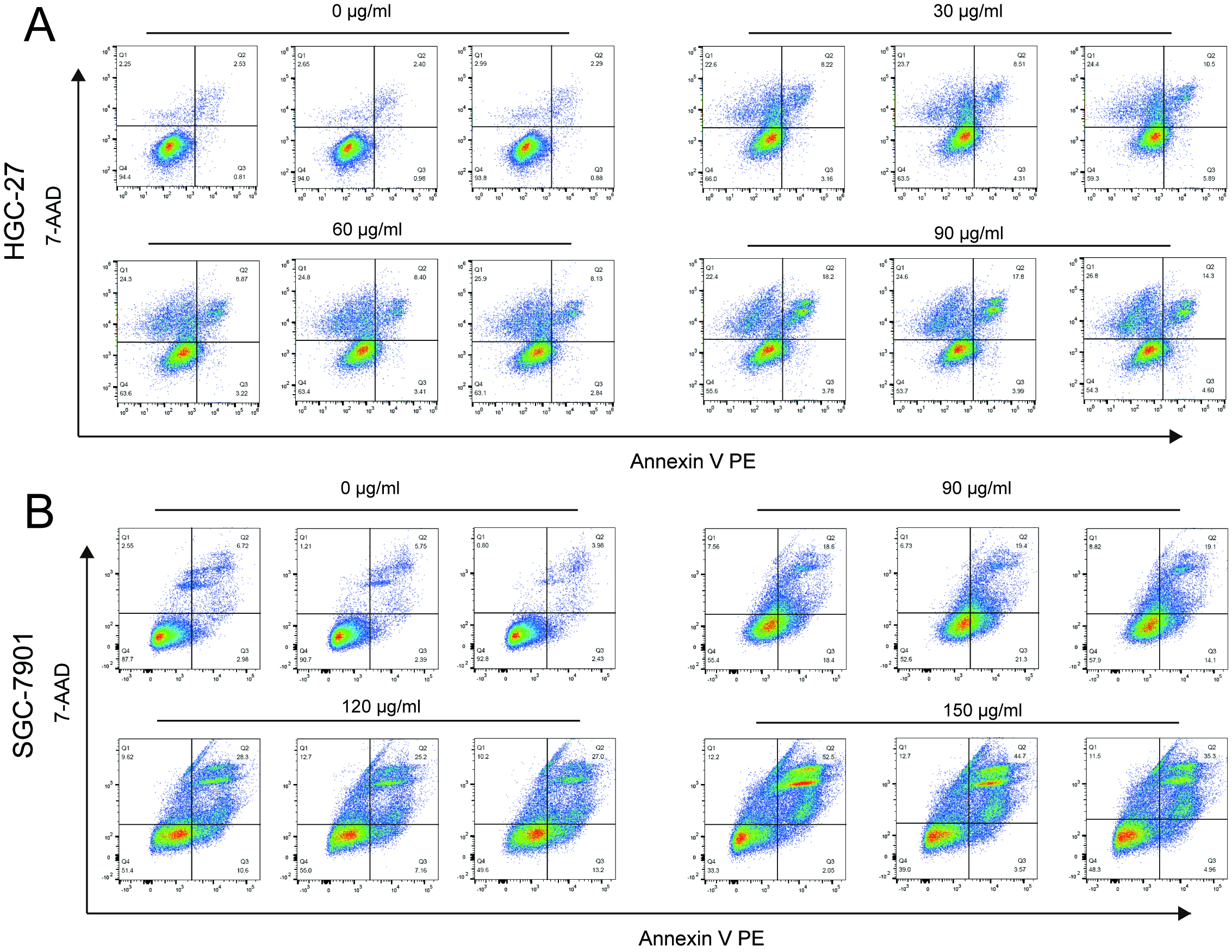

Supplement: Supplementary file 8 [file Image2.TIF]

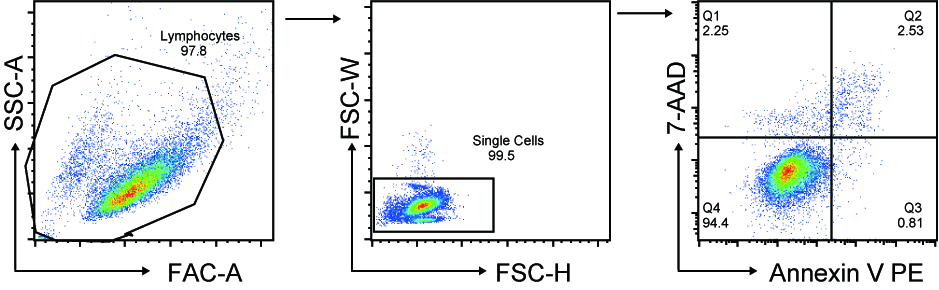

Supplement: Supplementary file 10 [file Image1.TIF]

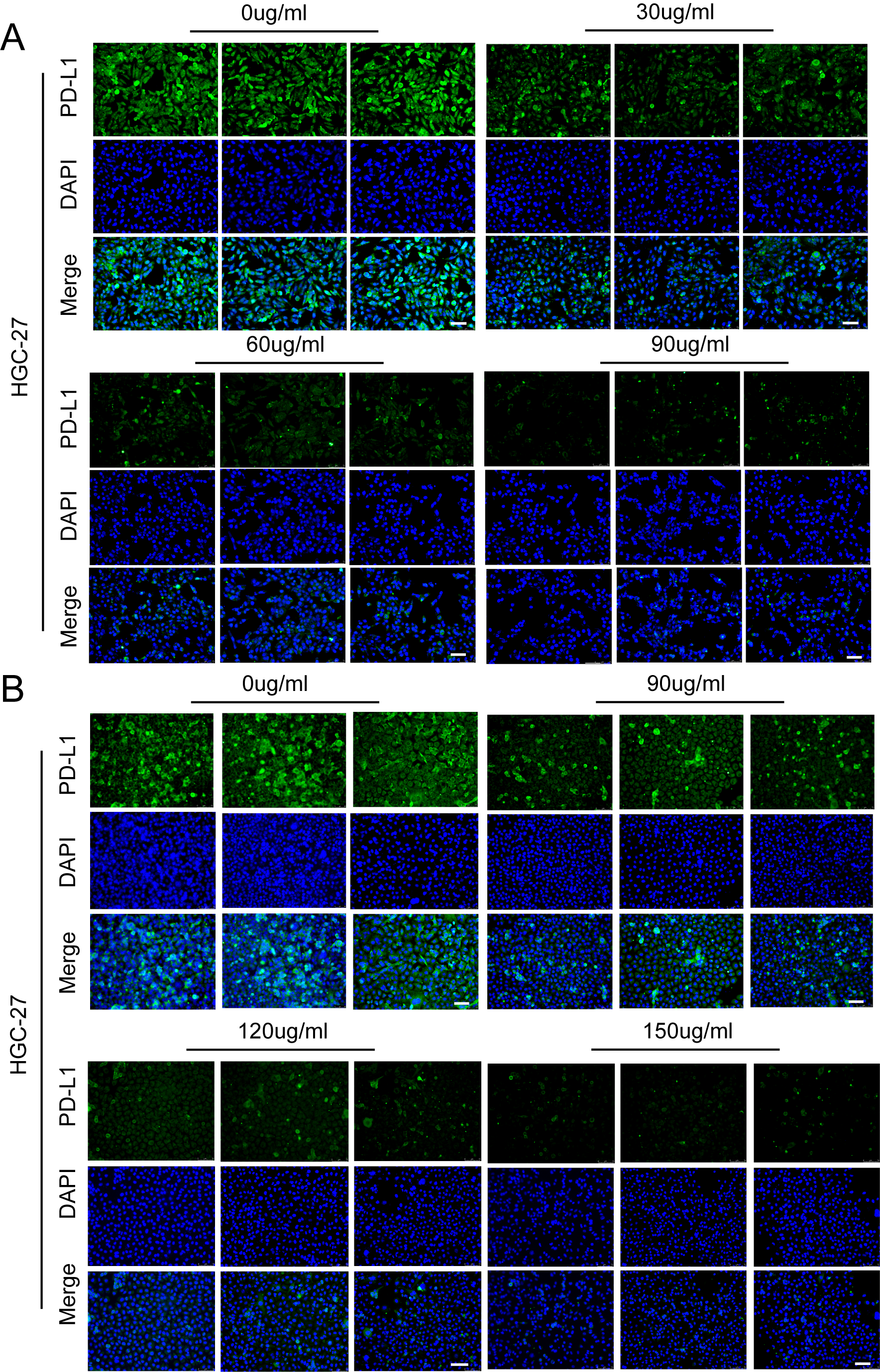

Supplement: Supplementary file 11 [file Image7.TIF]

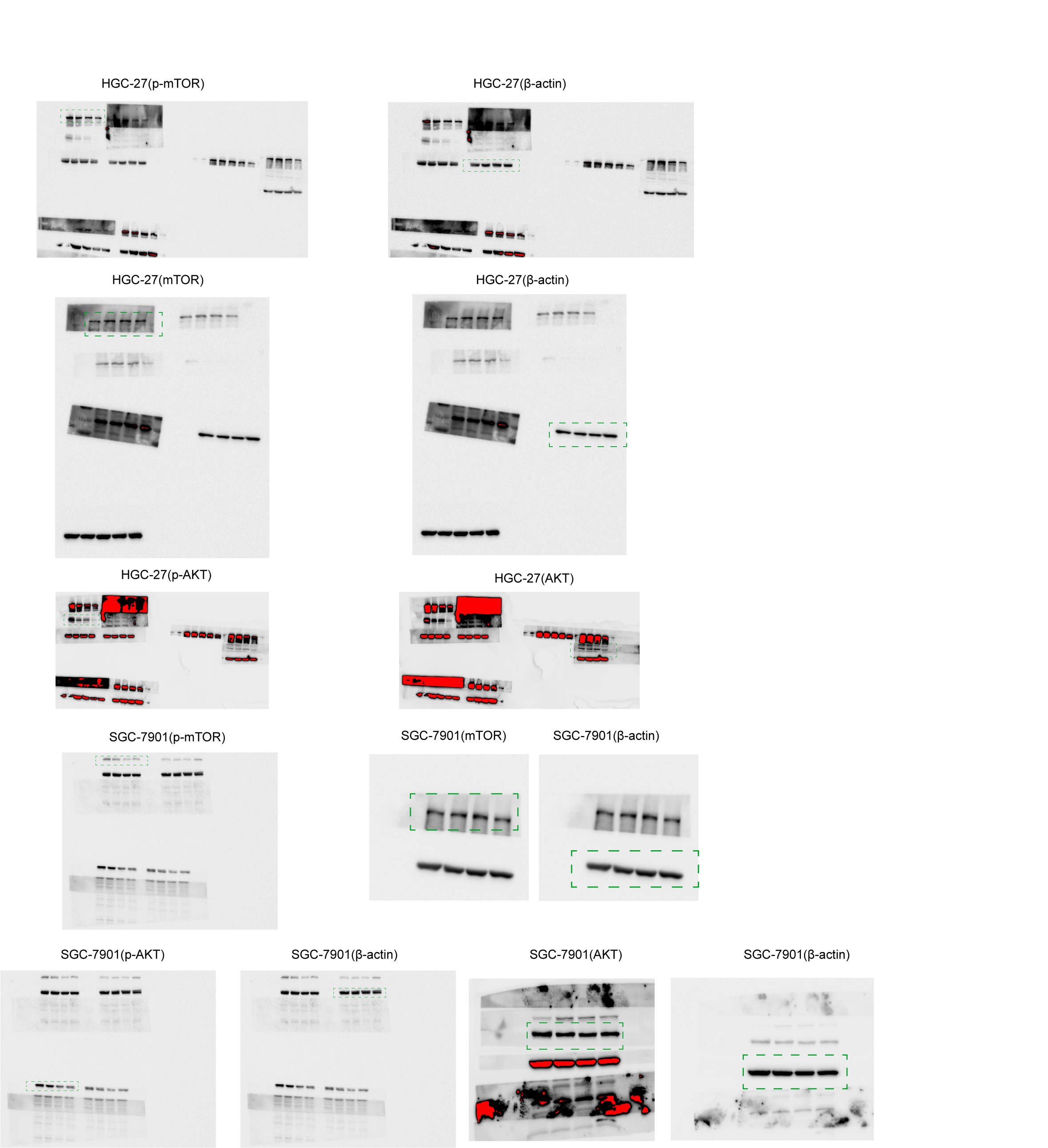

Supplement: Supplementary file 17 [file Image5.TIF]
