# Supplementary material for: Gypenoside induces apoptosis by inhibiting the PI3K/AKT/mTOR pathway and enhances T-cell antitumor immunity by inhibiting PD-L1 in gastric cancer
Source: Front Pharmacol. 2024 Feb 28;15:1243353. doi: 10.3389/fphar.2024.1243353 (PMC10933075; doi:10.3389/fphar.2024.1243353)
Supplement: Supplementary file 18 [file DataSheet1.docx]

Calcein AM-PI staining, PD-L1 staining images and flow cytometry data are upload to jianguoyun. Link as follows <https://www.jianguoyun.com/p/DfKtYlUQj7HoCxiH1ZEFIAA>.
